# Daily voluntary exercise enhances pilocarpine‐induced saliva secretion and aquaporin 1 expression in rat submandibular glands

**DOI:** 10.1002/2211-5463.12353

**Published:** 2017-12-07

**Authors:** Kentaro Matsuzaki, Naotoshi Sugimoto, Masanori Katakura, Eri Sumiyoshi, Toshiko Hara, Michio Hashimoto, Osamu Shido

**Affiliations:** ^1^ Department of Environmental Physiology Faculty of Medicine Shimane University Izumo Japan; ^2^ Department of Physiology Graduate School of Medical Science Kanazawa University Japan; ^3^ Department of Nutritional Physiology Faculty of Pharmaceutical Sciences Josai University Saitama Japan

**Keywords:** aquaporin, submandibular gland, vascular endothelial growth factor, voluntary exercise

## Abstract

Saliva—a water‐based fluid containing electrolytes, immunoglobulins, and enzymes—has many functions, including the protection and hydration of mucosal structures within the oral cavity and the initiation of digestion. Aquaporins (AQPs) are proteins that act as water channels through membranes. We have previously reported upregulation of the expression levels of AQP 1 and 5 in the submandibular glands (SMGs) in heat‐acclimated rats. In this study, we investigated pilocarpine‐induced saliva secretion and AQP expression in rats after voluntary exercise. Male, 10‐week‐old Wistar rats were initially maintained at an ambient temperature of 24 °C for 10 days and were then kept for 40 days in cages either with a running wheel (exercise rats, *n* = 6) or with a locked wheel [control rats (CN), *n* = 6]. After the training period, the rats were anesthetized and pilocarpine, an M3 muscarinic receptor agonist, was intraperitoneally injected (0.5 mg·kg^−1^) to stimulate saliva secretion. Saliva was collected, and the SMGs were sampled and subjected to western blot, RT‐PCR, and immunohistochemical analyses. Pilocarpine induced a greater amount of saliva in the exercised rats than in the CN. Expression levels of AQP1 mRNA and protein were significantly higher in SMGs of exercised rats than in those of the CN, but the expression of AQP5 was not affected by voluntary exercise. Voluntary exercise increased the expression of vascular endothelial growth factor (VEGF) and cluster of differentiation 31 (CD31), a marker for endothelial cells, in the SMGs. Voluntary exercise promoted pilocarpine‐induced saliva secretion, probably via an increase in the expression level of AQP1 due to VEGF‐induced CD31‐positive angiogenesis in the SMG.

**Abbreviations**
AQP: aquaporin
CD31: cluster of differentiation 31
CN: control rats
EX: exercised rats
SMG: submandibular gland
*T*_core_: core body temperature
VEGF: vascular endothelial growth factor

Salivary glands are involved in the secretion of saliva, which is known to participate in several processes, including protection and hydration of mucosal structures within the oral cavity, the initiation of digestion, antimicrobial defense, and protection from chemical and mechanical stress 1, 2. Saliva is a water‐based fluid containing electrolytes, immunoglobulins, and enzymes, and its secretion can be stimulated by muscarinic and adrenergic agonists. As water movement is involved in saliva secretion, aquaporins (AQPs), a family of transmembrane proteins that act as water channels, are present in salivary glands; the expression, localization, and function of AQPs have been studied 3. The presence of AQP1, AQP5, and AQP8 in salivary glands is generally accepted, but the presence of AQP3, AQP4, AQP6, and AQP7 remains controversial 4, 5, 6, 7, 8. AQP1 is known to be restricted to the endothelial cells of the microvasculature in salivary glands; it is involved in the transport of water from a blood vessel to the salivary gland cells across the basolateral membrane. AQP5 has been detected on the luminal side of acinar and duct cells 9, 10. Thus, AQP1 and AQP5 undeniably have important roles in salivary secretion. However, around 10% of the general population and 25% of elderly people have xerostomia, known as dry mouth syndrome, which is defined by a decrease in salivary function. A dry mouth significantly increases the risk of tooth decay and other oral dysfunctions such as dysphagia and stomatitis. Several factors may cause a persistently dry mouth, including prescription medications, medical treatments, and certain autoimmune diseases such as Sjögren's syndrome 2. Interestingly, AQP1 expression in labial salivary glands is selectively downregulated in patients with primary Sjögren's syndrome 11, and this decrease in AQP1 in the salivary gland may play a crucial role in the pathology of this disease. This suggests that the precise regulation of AQP1 expression in salivary glands may enhance the saliva secretary function.

Rodents can apply saliva to their body trunk as a substitute for sweat during increases in core body temperature (*T*_core_) or environmental temperature 12, 13. We reported previously that constant exposure to moderate heat increased the expression of AQP1 and AQP5 in the submandibular glands (SMGs) of rats concomitant with the promotion of the hypoxia‐inducible factor‐1α (HIF‐1α) pathway, leading to the upregulation of the vascular endothelial growth factor (VEGF), as well as cluster of differentiation 31 (CD31)‐positive angiogenesis 8. We hypothesized that the increased expression of AQP1 and AQP5 was the result of a rise in *T*_core_ due to heat exposure and that this enhanced the salivary secretory function. These findings suggest a further hypothesis, namely that an increase in *T*_core_ through voluntary exercise might also promote salivary secretion via enhancement of the expression of AQPs. The aim of this study therefore was to investigate changes in the expression levels of AQP1 and AQP5 in the SMGs of rats that underwent a period of voluntary exercise. Furthermore, distribution of AQP1, AQP5 VEGF, and CD31 in the SMGs was histologically investigated. We also measured saliva secretion induced by pilocarpine, an M3 muscarinic receptor agonist with a sympathetic‐like character 14, which has been shown to be useful for the assessment of the stress response on saliva secretion 15, 16.

## Materials and methods

### Ethics statement

All animal experiments were performed in accordance with the Guidelines for Animal Experimentation of the Shimane University Faculty of Medicine, compiled from the Guidelines for Animal Experimentation of the Japanese Association for Laboratory Animal Science. The protocol for this study was approved by the Committee on the Ethics of Animal Experiments of the Shimane University.

### Experimental schedule

Male, 10‐week‐old Wistar rats (Japan SLC Inc., Hamamatsu, Japan) were maintained for 10 days at an ambient temperature of 24 ± 0.1 °C and relative humidity of 45% ± 5% under a 12 : 12‐h light–dark cycle (lights on at 7:00 h), with food and water *ad libitum*. After this, the rats were assigned to one of two groups, exercise (EX, *n* = 6) and control (CN, *n* = 6). The EX rats were kept for 40 days in cages with a running wheel (SN‐451, Shinano Seisaku, Tokyo, Japan), allowing them to undertake voluntary exercise, while the CN rats were kept in cages with the running wheel locked. On the 40th day, pilocarpine‐induced saliva was measured as follows. Briefly, the rats were anesthetized, preweighed cotton was placed in their mouths sublingually, and pilocarpine (0.5 mg·kg^−1^; Wako, Tokyo, Japan) was intraperitoneally injected to induce saliva secretion. Each cotton ball was then changed every 10 min for 1 h. The collected cotton balls were weighed again, and the mass of saliva secreted was calculated by subtracting the initial from the final weight. After these measurements, the cotton balls were centrifuged and the saliva was collected. The Na^+^ concentration in the saliva was measured using a Horiba compact ion meter (Horiba, Tokyo, Japan) and the protein concentration using a BCA protein assay kit (Thermo Fisher Scientific, Waltham, MA, USA). After saliva collection, blood samples were transcardially collected and blood cell components were measured by KX‐21NV (Sysmex, Hyogo, Japan). Then, the SMGs of rats were sampled and the tissues weighed. After that, the SMGs were used for western blotting, RT‐PCR, and immunohistochemical analyses.

### Western blot analysis

The SMGs were homogenized using a glass homogenizer in lysis buffer including 1 mm EDTA, 1% SDS, 1× complete Protease Inhibitor Cocktail tablet (Roche Diagnostics, Switzerland), and 10 mm HEPES (pH 7.5). After sonication and removal of the tissue debris by centrifugation at 800 ***g*** for 15 min at 4 °C, the supernatants were analyzed by western blotting, as described previously 17. Briefly, proteins were extracted from the SMGs and the protein concentrations were determined using a protein assay kit. Equal amounts of protein were separated by 10% or 12.5% SDS/PAGE. The resolved proteins were transferred onto polyvinylidene fluoride (PVDF) membranes (Millipore, Billerica, MA, USA) blocked with 5% skimmed milk and then incubated with anti‐AQP1 antibody (1 : 1000; Abnova, Taipei, Taiwan), anti‐AQP5 antibody (1 : 1000; Calbiochem, La Jolla, CA, USA), anti‐VEGF antibody (1 : 1000; Thermo Fisher Scientific), anti‐CD31 antibody (1 : 1000; Gene Tex, San Antonio, TX, USA), and anti‐β‐actin antibody (1 : 2000; Cell signaling, Danvers, MA, USA). After washing, the PVDF membranes were incubated with horseradish peroxidase (HRP)‐linked secondary antibodies (1 : 2000; Cell Signaling). The blots were developed using Immobilon Western Chemiluminescent HRP Substrate (Millipore) and were visualized using an image analyzer (LAS‐4000; FUJI FILM, Tokyo, Japan). The membranes were then stripped and reprobed with monoclonal rabbit anti‐β‐actin antibody (1 : 2000; Cell Signaling) as described previously 17.

### Total RNA isolation and RT‐PCR analysis

To evaluate the mRNA expression of AQP1, AQP5, M1‐type muscarinic acetylcholine receptor (M1), M3‐type muscarinic acetylcholine receptor (M3), VEGF, and β‐actin in the SMGs, RT‐PCR analysis was performed as described previously 8. Briefly, the total RNA was isolated using an RNeasy Mini Kit (Qiagen, Valencia, CA, USA), and first‐strand cDNA was synthesized from 1 μg of total RNA using a High Capacity cDNA Reverse Transcription Kit (Applied Biosystems, Carlsbad, CA, USA). RT‐PCR analysis of AQP1, AQP5, VEGF, M1, M3, and β‐actin mRNA levels was performed with GoTaq (Promega, Madison, WI, USA) using the following primers: 5′‐catgtacatcatcgcccagt‐3′ and 5′‐ccacagccagtgtagtcaat‐3′ for AQP1; 5′‐gcccagctggtgggcgccatt‐3′ and 5′‐tggggagcccacagggctggt‐3′ for AQP5; 5′‐cctggctttactgctgtacct‐3′ and 5′‐gatgtccaccagggtctcaat‐3′ for VEGF; 5′‐cagcagcagctcagagaggtc‐3′ and 5′‐ggtgcctgtgcttcagaatct‐3′ for M1; 5′‐ccaagcttcccatccagttag‐3′ and 5′‐gtgttcaccaggaccatgatg‐3′ for M3; and 5′‐atggtgggtatgggtcagaag‐3′ and 5′‐ctggggtgttgaaggtctcaa‐3′ for β‐actin. The PCR products were electrophoresed using agarose gel, and the PCR bands were detected under ultraviolet light.

### Immunohistochemistry

The SMGs were fixed overnight in 10 N Mildform (Wako) at 4 °C and immersed in a 20% (w/v) sucrose solution. A cryostat was used to prepare 15‐μm‐thick SMG sections, which were then subjected to a 10 mm sodium citrate buffer (pH 6.0) and blocked with 3% normal goat serum. For multiplex immunoassaying, the SMG sections were incubated with several primary antibodies for 12 h at 4 °C. The primary antibodies used in this study were monoclonal mouse anti‐VEGF IgG (1 : 500; Cell Signaling) and monoclonal mouse anti‐CD31 IgG (1 : 200; Millipore). Alexa Fluor 488 anti‐mouse IgG (1 : 500; Molecular Probes, Eugene, OR, USA) was used as secondary antibody. To detect cell nuclei, sections were counterstained with DAPI (Dojindo, Tokyo, Japan). After staining, the sections were covered with 80% glycerol. A confocal microscope (FV‐1000D; Olympus, Tokyo, Japan) and imaging software (Fluoview; Olympus, Japan) were used to visualize all sections under 20× or 40× magnification, as described previously 18, 19. Alexa Fluor 488 and DAPI filters were used to observe the immunopositive cells.

### Data quantification and statistical analysis

The results are presented as the mean ± SEM. The parameters were analyzed by Student's *t*‐tests. Statistical analyses were performed with spss software version 18.0 (IBM Corp., Armonk, NY, USA). A *P* value < 0.05 was considered to indicate statistical significance.

## Results

### Wheel‐running activity and blood cell components

**Figure 1:**
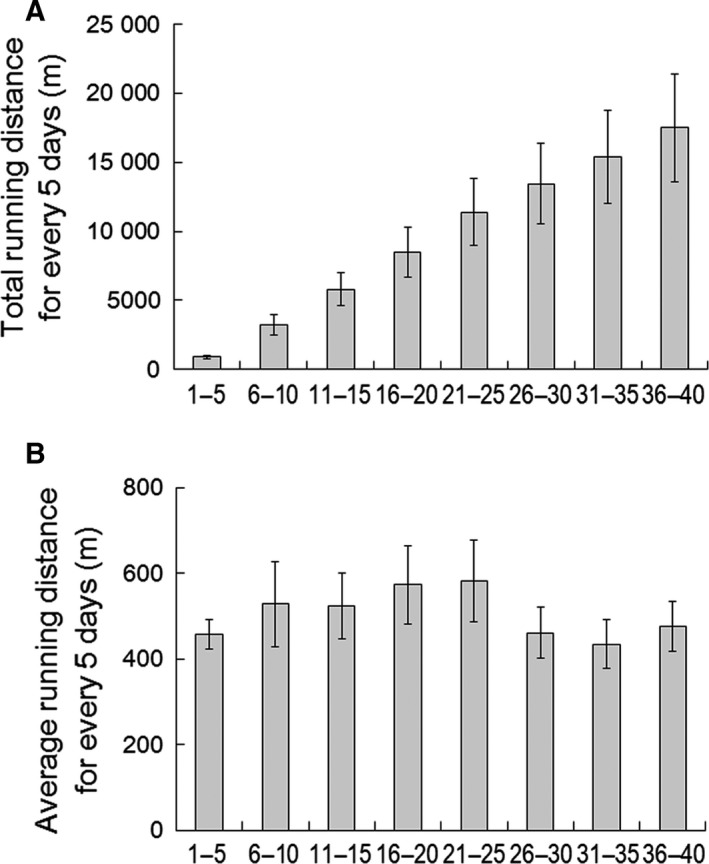
Distances run by the EX rats. (A) Total running distance for every 5 days and (B) average running distance for every 5 days. Values are means ± SEM (*n* = 6).

The total and average running distances for every 5 days of the EX rats are shown in Fig. 1A,B, respectively. The total running distances significantly increased over time (Fig. 1A, *P* < 0.0001). Average running distances of EX rats were stable during 40‐day experimental period (Fig. 1B). The blood cell components of the CN and EX rats are summarized in Table 1. There was no significant difference between the CN and EX rats for any blood cell component (Table 1).

**Table 1:** Blood cell components for the control (CN) and exercise group (EX) rats. WBC, white blood cell count; RBC, red blood cell count; HGB, hemoglobin; HTC, hematocrit; MCV, mean corpuscular volume; MCH, mean corpuscular hemoglobin; MCHC, mean corpuscular hemoglobin concentration; PLT, platelets. There was no significant difference in any blood cell component between the CN and EX rats

	CN		EX		*P* value
	Mean	SE	Mean	SE	
WBC (× 10^2^/μL)	71.71	3.23	67.50	3.55	0.401
RBC (× 10^4^/μL)	860.57	16.61	887.13	17.79	0.300
HGB (g·dL^−1^)	15.06	0.32	15.84	0.28	0.086
HTC (%)	47.27	1.25	49.86	0.93	0.116
MVC (fL)	54.93	1.07	55.43	1.06	0.747
MCH (pg)	17.49	0.18	17.86	0.29	0.303
MCHC (g·dL^−1^)	32.20	0.59	31.78	0.04	0.453
PLT (× 10^4^/μL)	68.39	2.45	62.70	4.84	0.335

### Pilocarpine‐induced saliva secretion

**Figure 2:**
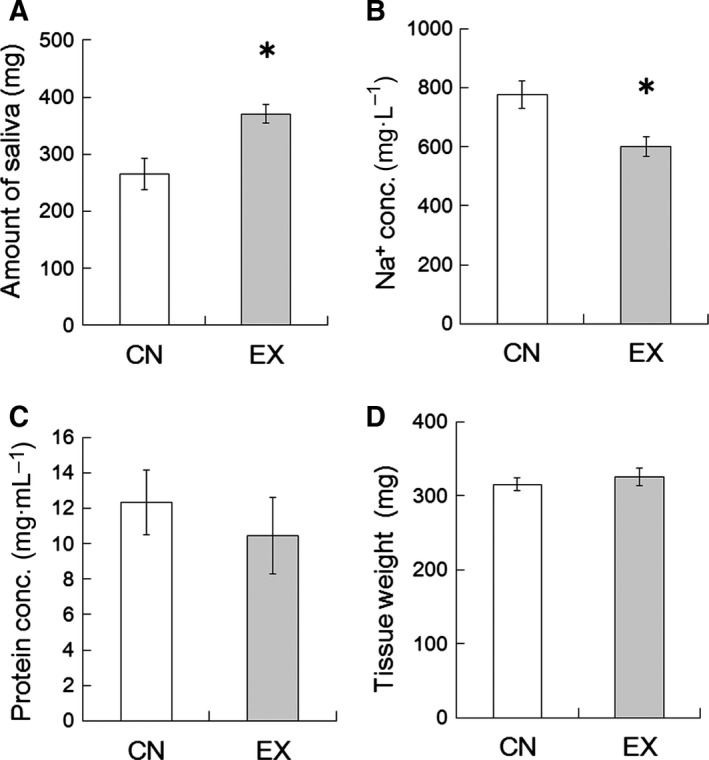
Amount of saliva, Na^+^ concentration, protein concentration, and SMGs tissue weight in the CN and EX rats. (A) Amount of pilocarpine‐induced saliva secreted. (B) Na^+^ concentration of the saliva, which was reduced by running wheel exercise. (C) Protein concentration in the saliva. (D) Tissue weights of the SMGs. The protein concentration and tissue weights were not changed by voluntary exercise. Values are means ± SEM (*n* = 6 in each group). *, significant difference between the CN and EX rats (*P* < 0.05).

We examined the pilocarpine‐induced saliva secretion of the CN and EX rats. A significantly greater amount of saliva was induced by pilocarpine in the EX rats than in the CN rats (Fig. 2A, *P* < 0.01). Conversely, the Na^+^ concentration in the saliva of the EX rats was significantly lower than that of the CN rats (Fig. 2B, *P* < 0.05). There was no significant difference between the CN and EX rats in the protein concentration of the saliva (Fig. 2C, *P* = 0.89) or the SMG tissue weight (Fig. 2D, *P* = 0.91).

### AQP1 and AQP5 expression in the SMGs

**Figure 3:**
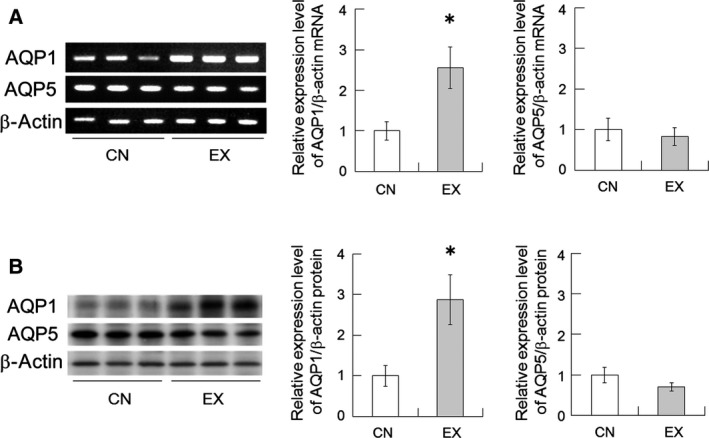
AQP1 and AQP5 expression in the SMGs of CN and EX rats. (A) AQP1 and AQP5 mRNA expression. (B) AQP1 and AQP5 protein expression. Running wheel exercise increased the expression of AQP1 mRNA and protein in the SMGs. Values are the means ± SEM (*n* = 6 in each group). *, significant difference between CN and EX rats (*P* < 0.05).

The mRNA expressions of AQP1 and AQP5 were detectable in the CN and EX rats. The mRNA expression of AQP1 was found to be significantly increased in the EX rats in comparison with the CN rats (Fig. 3A, *P* < 0.05), whereas AQP5 mRNA levels were not changed (Fig. 3A). To confirm the results of RT‐PCR, we examined the expression of AQP1 and AQP5 protein in the SMGs by western blotting using anti‐AQP1 and anti‐AQP5 antibodies. As expected, AQP1 protein levels increased in the EX rats after 40 days of voluntary exercise (Fig. 3B, *P* < 0.05), whereas AQP5 protein levels were not altered (Fig. 3B). These results suggest that voluntary running exercise may regulate the expression of AQP1 in rat SMGs. Additionally, we measured AQP5 protein levels in saliva by western blotting, because AQP5 is released into saliva and salivary AQP5 levels correlate with salivary secretion 20. However, salivary AQP5 was not detected in the saliva collected from either the CN or the EX rats (data not shown).

### VEGF and CD31 expression in the SMGs

**Figure 4:**
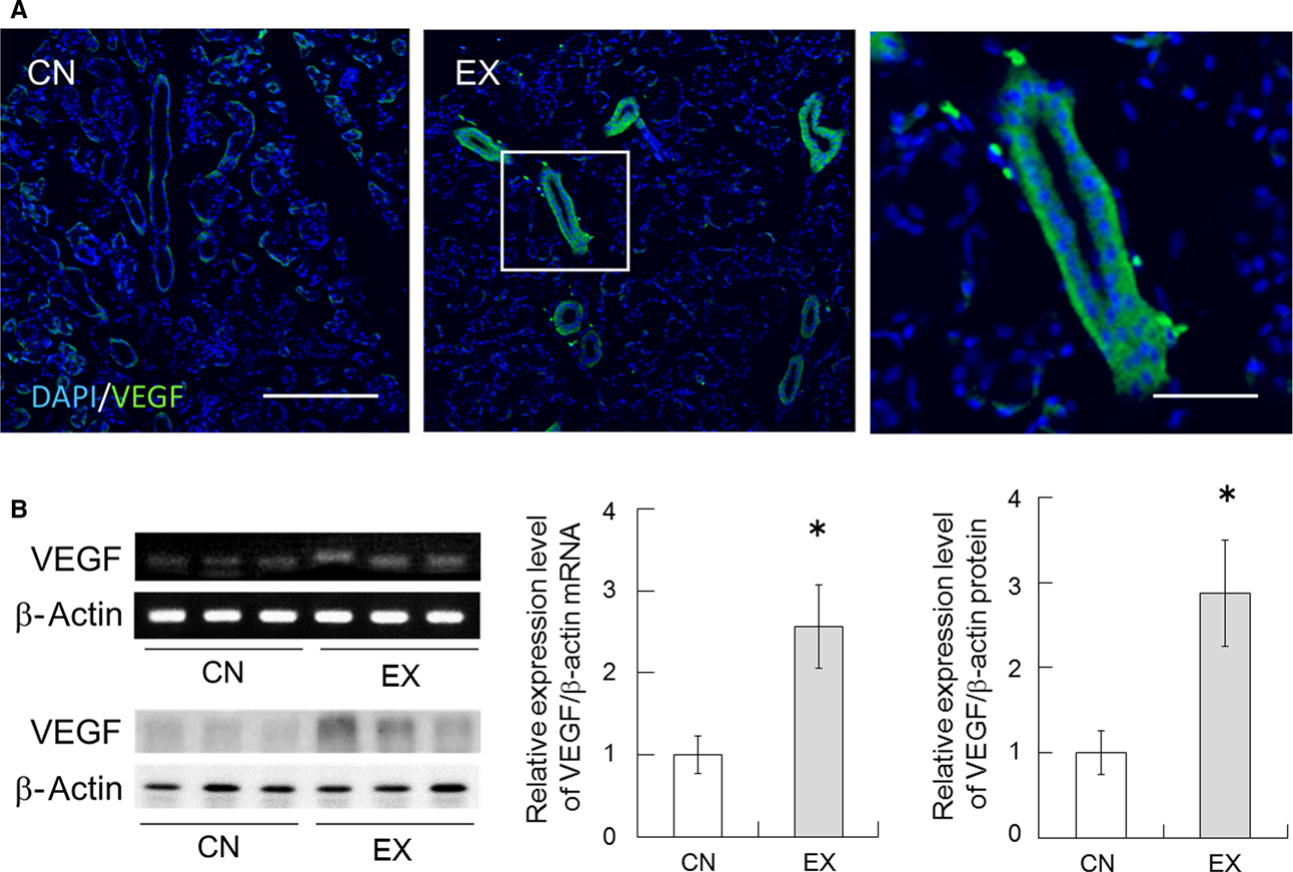
VEGF expression in the SMGs of CN and EX rats. (A) Immunohistochemical analysis of VEGF (green) in the SMGs. The nuclei were stained with DAPI (blue). Scale bar; 100 μm. The right panel shows magnified views of the boxed regions from the EX rats. Scale bar, 25 μm. (B) VEGF mRNA and protein expression. Running wheel exercise increased the expression of VEGF in SMGs. Values are means ± SEM (*n* = 6 in each group). *, significant difference between the CN and EX rats (*P* < 0.05).

To test whether VEGF was involved in the changes resulting from voluntary exercise, we examined the expression of VEGF mRNA and protein in the SMGs. The immunohistochemical analysis detected VEGF protein in the serous acinar cells, duct cells, and capillaries of the SMGs, as described previously 21, and showed that it was upregulated in the EX rats (Fig. 4A). VEGF mRNA expression was detectable in both the CN and the EX rats, with the levels increased in the EX rats (Fig. 4B, *P* < 0.05). To confirm the results of RT‐PCR, we examined the expression of VEGF protein in the SMGs by western blotting, which showed that it was significantly upregulated in the EX rats (Fig. 4B, *P* < 0.05). These results suggest that a period of voluntary exercise may regulate the expression of VEGF to induce angiogenesis in the SMGs of rats.

**Figure 5:**
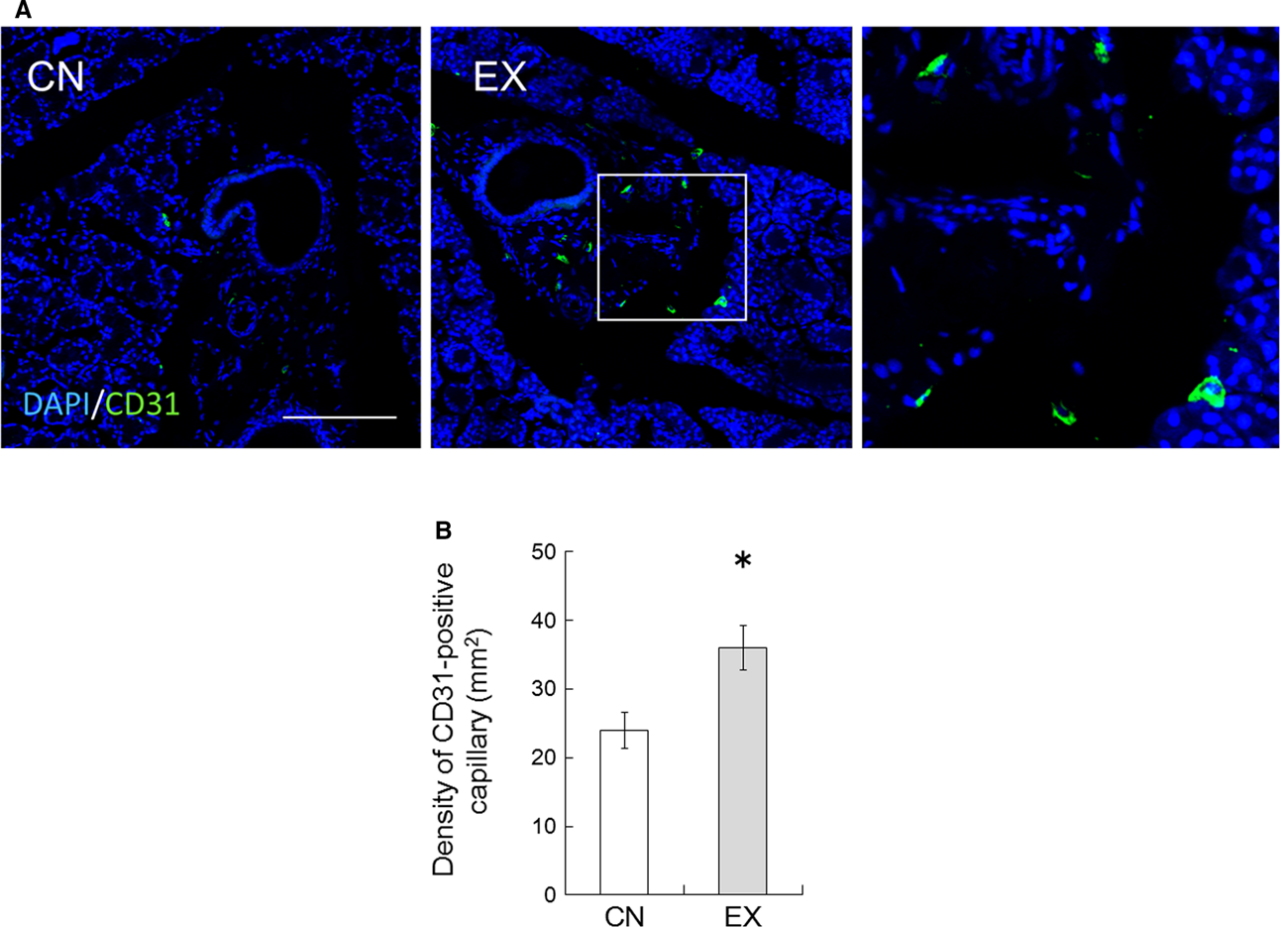
CD31 expression in the SMGs of CN and EX rats. (A) Immunohistochemical analysis of CD31 (green) in the SMG. The nuclei were stained with DAPI (blue). Scale bar, 100 μm. The right panel shows magnified views of the boxed regions from the EX rats. Scale bar, 25 μm. (B) Density of capillaries in the SMG sections. The densities of CD31‐positive capillaries were counted in randomly chosen high‐power fields per section and expressed as the number of capillaries per mm^2^. Values are means ± SEM. *P < 0.05. Running wheel exercise increased the expression of CD31 in SMGs. Values are means ± SEM (*n* = 6 in each group). *, significant difference between CN and EX rats (*P* < 0.05).

We also investigated the expression level in the SMGs of CD31, a marker for vascular endothelial cells. The immunohistochemical analysis detected CD31 in endothelial cells and in a few stroma cells that showed cytoplasmic staining (Fig. 5A). The number of CD31‐positive capillaries was counted for randomly chosen high‐power fields in each tissue and expressed as the number of capillaries per mm^2^. CD31‐positive capillaries in the SMGs increased in the EX rats compared with the CN rats (Fig. 5B, *P* < 0.05), indicating that voluntary exercise may promote an increase in capillaries in the SMGs.

### M1 and M3 receptor expression in the SMGs

**Figure 6:**
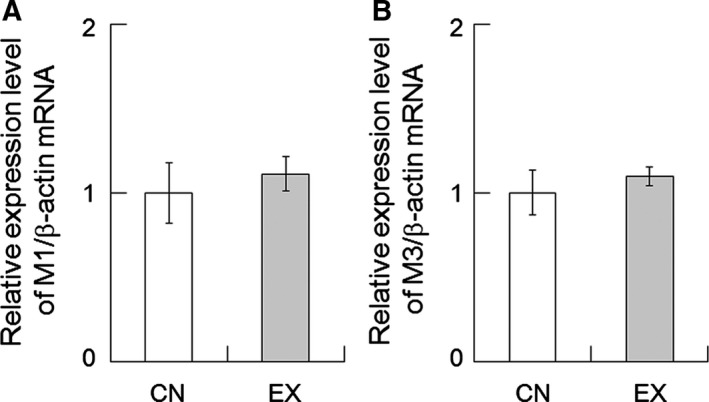
M1 and M3 muscarinic receptor mRNA expression in the SMGs of CN and EX rats. (A) M1 and (B) M3 mRNA expression in the SMGs. There were no significant differences in M1 and M3 expression between the CN and EX rats. Values are means ± SEM (*n* = 6 in each group).

We examined the expression levels of M1 and M3 muscarinic receptor mRNA in the SMGs by RT‐PCR. M1 and M3 muscarinic receptor expressions were detectable in both CN and EX rats. However, the mRNA expression of M1 and M3 had not changed after 40 days of voluntary exercise (Fig. 6).

## Discussion

Voluntary exercise increased pilocarpine‐induced saliva secretion in rats (Fig. 2A). This result suggests the possibility that water permeability may have enhanced in the SMGs of the rats that underwent voluntary exercise. The saliva is composed of a variety of electrolytes (such as Na^+^), immunoglobulins, mucus, glycoproteins, enzymes, antimicrobial agents and lysozyme, and other enzymes; it is a very dilute fluid composed of more than 99% water 2. In this study, Na^+^ concentration in the secreted saliva decreased significantly in the EX rats (Fig. 2B). Moreover, the protein concentration of saliva in the EX rats tended to decrease compared with the values for the CN rats (Fig. 2C), but the SMG tissue weight remains unchanged (Fig. 2D). As water permeability of the SMGs was increased by exercise training, Na^+^ and protein concentration in secreted saliva may have been relatively decreased. It is well known that water channels AQP1 and AQP5 are important for water permeability on the salivation, which is why their expression levels in the SMGs were investigated in this study. The expression of AQP1 was found to be significantly increased in the SMGs of the EX rats (Fig. 3). This upregulation of the EX rats may have been involved, at least partially, in the increased salivation. In rat salivary glands, AQP1 expression is restricted to the endothelial cells of the microvasculature, suggesting that AQP1 contributes in the microvasculature to the permeability of water from plasma across the endothelial barrier 5, 10, 22. We therefore inferred the effects of voluntary exercise on blood flow in the SMG, because blood flow is an important factor influencing secretion from endocrine glands 23. Angiogenesis usually results in an increase in tissue blood flow 24. VEGF is a signal protein produced by cells that stimulates vasculogenesis and angiogenesis 25, 26. VEGF mRNA and protein are expressed in the SMGs 8, 27 and have an important role in the maintenance of the homeostasis of mucous membranes, with the induction of angiogenesis helping to accelerate wound healing within the oral cavity 27. In addition, it has been reported that VEGF in the salivary gland permeabilizes intraglandular capillaries and participates in the regulation of saliva production 27. CD31 is known to be a platelet endothelial cell adhesion molecule (PECAM‐1) and is normally found on endothelial cells, platelets (PLT), macrophages and Kupffer cells, granulocytes, lymphocytes, megakaryocytes, and osteoclasts 28. CD31 is known to have various roles in vascular biology, including angiogenesis; for example, CD31 appears to be required for cell elongation, migration, and/or invasion as well as for cell–cell association to form the endothelial structures 29, 30. In the present study, the expression levels of the potent angiogenic factors VEGF and CD31 were upregulated in the SMGs of the EX rats (Figs 4 and 5). These results suggest that voluntary exercise promotes salivary function via an increase in the expression of AQP1 due to VEGF‐induced CD31‐positive angiogenesis in the SMG.

In contrast, AQP5 expression in the SMG was not enhanced by voluntary exercise. AQP5 is restricted to the apical membrane of acinar cells, where it allows fluid to flow out into the acinar lumen and it plays a major role in the rapid movement of water in salivary glands 3, 10, 31. Previously, we reported that chronic exposure to moderate heat (32 °C) enhances the expression of both AQP1 and AQP5 in rat SMGs 8. Although the exact reason for this difference in results is unknown, it may be related in part to the duration of the increase in *T*_core_. We have previously measured *T*_core_ of voluntary exercised rats 32. *T*_core_ increased during each bout of spontaneous running. However, each rise of *T*_core_ was apparently followed by a profound hypothermia. Such postexercise hypothermia was also evident in the report of Satinoff *et al*. 33 showing a marked rise in the level of *T*_core_ during the period when rats were allowed access to a running wheel. On the other hand, heat exposure throughout the day raises *T*_core_ chronically 18, 34. These differences between voluntary exercise and heat exposure may be at least partly related to the expression level of AQP5. The changes in AQP5 expression level, however, may be involved in not only body temperature but also other factors, such as locomotor activity, plasma osmotic pressure, food and water intake, metabolic rate, and oxygen consumption. The relationship between the duration of changes in physiological factors and the expression of AQP5 should be examined in future studies.

It has been reported that AQP8 is expressed in the salivary glands, although there are discrepancies between the reports. Koyama *et al*. 35 demonstrated the expression and localization of AQP8 mRNA in the acinar cells of rat salivary glands by *in situ* hybridization. In the present study, we were able to detect AQP8 mRNA in rat SMGs by RT‐PCR analysis with specific primers for this. However, exercise did not affect the expression of AQP8 mRNA in the SMG, as it did the AQP1 mRNA levels (data not shown).

In this study, the expression levels of AQPs, VEGF, and CD31 were examined after treatment with pilocarpine. We therefore confirmed whether pilocarpine has facilitatory effect on those expressions. However, pilocarpine itself did not alter the expression levels of those molecules in the SMGs (data not shown).

To ascertain whether the increase in pilocarpine‐induced saliva secretion was mediated by the increased expression of the muscarinic receptors, the expression levels of M1 and M3 mRNA were measured by RT‐PCR. These were not changed by voluntary exercise (Fig. 6). These results suggest that the increased salivary secretion induced by pilocarpine in the EX rats was not mediated by the upregulation of muscarinic receptors in the SMGs.

## Conclusions

The results of this study demonstrated that pilocarpine‐induced salivation is enhanced in rats by chronic exercise and that daily exercise increased the expression of AQP1 that may be endothelial cells at the capillaries. As AQP1 probably plays a crucial role in saliva secretion in humans, these findings may lead to a novel strategy for treating xerostomia such as that experienced in Sjögren's syndrome.

## Author contributions

KM, NS, and OS conceived the study. KM and ES performed the animal rearing. KM participated in the histological and biological analyses. KM prepared the figures and drafted the manuscript. KM, NS, MK, TH, ES, MH, and OS edited and revised the manuscript. All authors read and approved the final manuscript.
